# Acetate metabolic requirement of avian pathogenic *Escherichia coli* promotes its intracellular proliferation within macrophage

**DOI:** 10.1186/s13567-019-0650-2

**Published:** 2019-05-02

**Authors:** Xiangkai Zhuge, Yu Sun, Min Jiang, Juanfang Wang, Fang Tang, Feng Xue, Jianluan Ren, Weiyun Zhu, Jianjun Dai

**Affiliations:** 1 MOE Joint International Research Laboratory of Animal Health and Food Safety, College of Veterinary Medicine, Nanjing Agricultural University, Nanjing, China; 2 Key Lab of Animal Bacteriology, Ministry of Agriculture, Nanjing Agricultural University, Nanjing, 210095 China; 3 China Pharmaceutical University, Nanjing, 211198 China; 4 Center for Post-doctoral Studies of Veterinary Medicine, College of Veterinary Medicine, Nanjing Agricultural University, Nanjing, 210095 China; 5 Center for Post-doctoral Studies of Animal Husbandry, College of Animal Science & Technology, Nanjing Agricultural University, Nanjing, 210095 China

## Abstract

Avian pathogenic *Escherichia coli* (APEC) is a facultative intracellular pathogen, and intracellular persistence in macrophages is essential for APEC extraintestinal dissemination. Until now, there is still no systematic interpretation of APEC intracellular proliferation. Intracellular survival factors, especially involved in pathometabolism, need to be further revealed. Acetate plays critical roles in supporting energy homeostasis and acts as a metabolic signal in the inflammatory response of eukaryotes. In this study, we identified that APEC *acs*-*yjcH*-*actP* operon, encoding acetate assimilation system, presented the host-induced transcription during its proliferation in macrophages. Our result showed that this acetate assimilation system acted as a novel intracellular survival factor to promote APEC replication within macrophages. Furthermore, deletion of *acs*-*yjcH*-*actP* operon in APEC decreased its cytotoxic level to macrophages. qRT-PCR results showed that the production of pro-inflammatory cytokines (IL-1β, IL-6, IL-8, IL-12β, and TNF-α) and iNOS in FY26∆*acs*-*yjcH*-*actP* infected macrophages were obviously down-regulated compared to that in wild-type FY26 infected cells. Deletion of *actP*/*yjcH*/*acs* genes attenuated APEC virulence and colonization capability in avian lungs in vivo for colibacillosis infection models. And acetate assimilation system acted as a virulence factor and conferred a fitness advantage during APEC early colonization. Taken together, our research unravelled the metabolic requirement of APEC intracellular survival/replication within macrophages, and acetate metabolic requirement acted as an important strategy of APEC pathometabolism. The intracellular acetate consumption during facultative intracellular bacteria replication within macrophages promoted immunomodulatory disorders, resulting in excessively pro-inflammatory responses of host macrophages.

**Electronic supplementary material:**

The online version of this article (10.1186/s13567-019-0650-2) contains supplementary material, which is available to authorized users.

## Introduction

The intracellular infection of pathogenic bacteria causes metabolic adaptation responses to exploit nutrient or energy source of host cells, aiming to enhance the active survival and proliferation within host intracellular compartments [1–3]. The growing evidences show the pathogenic bacteria can regulate the expression of its metabolic pathways or virulence factors to deprive the necessary nutrients of the host cells, accompanied by host metabolic disorders as well as dysregulated antimicrobial reactions [2, 4, 5]. Such adaptations lead to changes in the metabolism between internalized bacteria and host cells, and the final outcome links with the pathogenicity [4, 6, 7]. Therefore, the phenomenon that the complex metabolic interactions during bacterial intracellular infection is termed as “pathometabolism” [1]. Pathometabolism has gradually become a research hotspot, and researchers attempt to reveal critical intracellular metabolic pathways and achieve a deeper understanding of bacterial infection [4, 7].

The intracellular bacteria can successfully survive and replicate within phagocytosis or non-phagocytosis cells, and also grow extracellularly in host body fluid environment or laboratory culture conditions [8]. Typical facultative intracellular bacteria, including *Mycobacterium tuberculosis*, *Salmonella typhi*, *Brucella*, *Legionella pneumophila*, and *Yersinia pestis*, replicate in specific bacteria-containing vacuoles (PCVs) within infected host cells [1]. The other pathogens, including *Shigella*, *Listeria*, Group A *Streptococcus*, and *Neisseria meningitides*, can escape from phagolysosomes into the cytosol of infected host cells where intracellular bacteria efficiently replicate [1, 9]. The cytosolic host nutrients can be directly acquired by the cytosolically replicating pathogens, whereas the intracellular bacteria residing in PCVs obtain the nutrients by transporters located in vacuolar membranes [1, 10, 11]. It seems that there is an intimate coordination between the metabolic systems and so-called “intracellular survival factors” of the intracellular pathogens, and the relationship is critical for the various stages of intracellular infection, such as replication within host cell compartments and reinfection of new host cells [12, 13]. Recent reports reveal that the metabolic activities of intracellular bacteria are essential for its resistance to the antimicrobial defense of host cells. Current research is still far from clear elucidation of the bacterial metabolism during its intracellular infection.

Macrophages play critical roles in host defense responses against microbial pathogens and act as the front line of host cells to encounter the pathogenic bacteria [4]. The successful pathogen must develop several mechanisms to hijack and overcome (at least partially) these antimicrobial responses [14, 15]. Moreover, it is not surprising that intracellular bacteria exploit and manipulate host nutrient metabolism as part of their intracellular survival and replication strategies [16]. Many studies show that intracellular replication of bacteria is closely linked to lipid metabolism of macrophages and perturbations of lipid metabolism, such as fatty acids and cholesterol, to prevent immune defenses and cellular functions of the infected macrophages [4, 7].

Acetate is one of the metabolic end products for glucose metabolism in enterobacteria. When growing aerobically, glucose is assimilated and first conversed into pyruvate through the glycolytic pathway, and the pyruvate is further transferred to acetyl-CoA by oxidative decarboxylation [17]. Then acetyl-CoA, so-called activated acetate, is conversed into acetate or acetic acid through acetyl-phosphate, including two-step reactions via phosphotransacetylase (Pta) and acetate kinase (AckA), encoded by *E. coli ackA*-*pta* operon [18]. In turn, acetate can be used as the sole carbon source of *E. coli* and other microbiota [17–20]. Under low concentration in the natural environment, acetate can be recruited by *E. coli* via specific membrane transporters. Two membrane transporters in *E. coli* are closely connected to trans-membrane transportation of acetate. The *actP* gene, variously named *yjcG*, encodes an acetate permease [20]. The *satP* gene in *E. coli* encodes a succinate-acetate transporter [21]. Single deletion of *satP* or *actP* genes in *E. coli* leads to a partial decrease in acetate uptake while double deletion of *satP* and *actP* genes almost abolishes *E. coli* acetate uptake activity [17, 21]. The *actP*, *yjcH* (encoding a hypothetical membrane protein), and *acs* (encoding acetyl-CoA synthetase, Acs) genes comprise one operon, highly conserved in *E. coli*. Bacteria can use high-affinity Acs to assimilate acetate as a carbon source under low concentrations. Acs catalyzes the conversion of intracellular acetate to acetyl-CoA by an irreversible reaction in *E. coli*, and acetyl-CoA enters central metabolism, such as TCA and glyoxylate cycle [17, 18].

It is well-known that *E. coli* lives in the intestine of warm-blooded animals as a harmless commensal strain. Due to loss or gain of mobile virulence-related islands and genetic elements, *E. coli* evolves into two major pathogenic categories: IPEC (Intestinal pathogenic *E. coli*) and ExPEC (Extra-intestinal pathogenic *E. coli*) [22–24]. IPEC harbors a series of intestinal virulence factors to cause intestinal and diarrheal diseases [24]. However, ExPEC holds an enhanced capability to cause infection in specific extraintestinal niches, such as colonizing in the human urinary tract, avian respiratory system, the bloodstream, and central nervous system [22, 25, 26]. Based on the different hosts and syndromes, ExPEC pathotypes are designated as avian pathogenic *E. coli* (APEC), uropathogenic *E. coli*, sepsis-associated *E. coli* (SEPEC), and neonatal meningitis *E. coli* (NMEC). In recent years, researchers have gradually recognized that APEC/ExPEC is a primary pathogen and acts as a major cause of the high-incidence of bacterial diseases [27–29]. APEC/ExPEC can evade the host immune barrier and escape from macrophage clearance into extraintestinal niches. APEC/ExPEC can utilize a variety of intracellular survival factors to successfully persist and replicate within infected phagocytosis cells [13]. Our previous studies indicate that ColV plasmids undertake critical function with APEC survival in macrophages. The two-component system PhoP/PhoQ controls HlyF (the ColV plasmid-encoded protein) to facilitate APEC escaping from phagolysosomes into the cytosol of infected macrophages [30].

The intracellular metabolic strategies utilized by APEC to enhance proliferative capacity within host phagocytosis cells remain poorly understood. In this study, owing to the comprehensive analysis of APEC acetate metabolic requirement, we revealed new identification of APEC pathogenic mechanism to promote its intracellular proliferation. Our results provided novel insights into metabolic crosstalk between pathogens and macrophages.

## Materials and methods

### Strains and plasmids construction

The description of the strains, plasmids, and PCR primers was shown in Additional file 1 and Additional file 2. The highly virulent APEC strains (FY26 and CVCC249) and human ExPEC strain RS218 acted as typical models to identify the molecular pathogenesis of APEC/ExPEC [30, 31]. The construction of the single *acs*, *actP*, *satP*, and *acs*-*yjcH*-*actP* operon mutants in wild-type FY26 was based on the Red recombinase method as the previously described [32]. The *acs*-*yjcH*-*actP* operon or the *satP* gene (containing the predicted promoters) was ligated in pSTV28 (a medium-copy plasmid, TaKaRa) to construct the complemented plasmid [30]. The complemented strains FY26C*acs*-*yjcH*-*actP* and FY26C*satP* were constructed by electroporating the complemented plasmids into the mutants, respectively.

### Cell culture

HD11 cells, Chicken macrophage-like line, were cultured with RPMI 1640 media (supplemented with 10% heat-inactivated fetal bovine serum, FBS) in a humidified atmosphere for 41 °C and 5% CO_2_. The RAW264.7 cells were cultured with DMEM media (10% FBS) at a 37 °C in a humidified incubator of 5% CO_2_. DF-1 cells, the chicken embryo fibroblast cell line, were cultured with DMEM media (10% FBS) at a 41 °C in a humidified incubator of 5% CO_2_. The mixed cancer HEp2 cells were maintained with DMEM media (10% FBS) at a 37 °C in a humidified incubator of 5% CO_2_.

### RNA isolation and quantitative real-time reverse transcription PCR

The total RNA was extracted from bacteria cultured in Luria–Bertani (LB) and isolated from blood for infected duck using an E.Z.N.A. bacterial RNA kit (Omega Bio-Tek, Beijing, China) according to the manufacturer’s protocol. The total RNA was treated with DNase I (Vazyme Biotech) for 1 h to remove the genomic DNA. To confirm free from contaminating DNA, PCR was conducted by the templates for RNA samples without reverse transcription.

For the total bacterial RNA extracted from the infected cells during bacteria infecting macrophages (HD11 and RAW246.7) and non-phagocytic cells, the monolayer cells were incubated with bacteria at an infection ratio 1:200 with 1 h, and the infected cells were treated with antibiotic [13]. The cells were harvested at 4 hpi or 8 hpi, and the cells were washed with PBS for three times. Then TRIZOL (invitrogen) was added to wells to lyse cells and extract the total RNA from the infected cells. In order to enrich bacterial mRNA, the total RNA from the infected cells was treated with MICROB*Enrich*™ Kit (Ambion; catalog no. AM1901) to remove host RNA [33, 34], and then processed with MICROB*Express*™ (Ambion; catalog no. AM1905) to deplete the bacterial rRNA [33, 34]. The each sample was repeated the above processes. The real-time PCR was conducted as previously described [33], and the primers for qRT-PCR were shown in Additional file 2. qRT-PCR data from three individual experiments were used to measure the differences (fold-change) of these genes transcription. The transcription level of the housekeeping gene *dnaE* acted as a reference to determine the expression level of targeted genes with the ∆∆*C*_*T*_ method as the previously described [35, 36]. qRT-PCR was conducted with the AceQ qPCR SYBR Green Master Mix (Vazyme Biotech) according to the manufacturer’s instruction.

Transcriptional level of selected genes for iNOS, pro-inflammatory cytokines (IL-1β, IL-6, IL-8, IL-12β, and TNF-α), and anti-inflammatory cytokines (IL-4, IL-10, and IL-13) were quantified [15, 37–39], and the corresponding primers were showed in Additional file 2. HD11 cells infected with APEC/ExPEC strains were extracted using TRIZol^®^ reagent (Invitrogen) at 4 hpi or 8 hpi. qRT-PCR was performed by SYBR^®^ *PremixEx* Taq™ (TaKaRa) to determine mRNA transcription of these cytokines. The gene transcription level was normalized to the gene *β*-*actin* with the ΔΔ*C*_*T*_ method [15, 37, 38].

### Growth curve

For the growth experiments in Luria-Bertani (LB) broth, wild-type FY26, mutants, and complemented strains were cultured over a course of 12 h at 37 °C, starting at 1.0 × 10^7^ CFU/mL. The cultured (200 μL) for 1 h apart were dropped to a 96-well plate in triplicate. A microplate reader was used to monitor the bacterial growth at an optical density OD_600_. The growth curve for each strain was obtained by averaged data from each read. The growth experiments in M9 minimal medium (carbon-source free) or plus acetic acid (60 mM, pH 6) were performed according to the previously described [21]. *E. coli* strains were cultured over a course of 36 h at 37 °C, starting at 5.0 × 10^7^ CFU/mL (OD_600_ = 0.1). Growth curve assay was performed at least three individual experiments.

### Cytokines ELISA assay

The monolayer cells were incubated with bacteria at a multiplicity of infection (MOI) of 10 with 1 h. Then the supernatant of infected cells with three replicate wells was harvested at 8 hpi and 16 hpi. The concentrations of cytokines IL-1β, IL-6, IL-8, IL-12β and TNF-α in the supernatant of RAW264.7 cells were measured using the corresponding cytokine ELISA kits, including mouse IL-1β ELISA Kit (Abcam, ab197742), mouse IL-6 ELISA Kit (Abcam, ab100712), mouse IL-8 ELISA Kit (Sangon Biotech, C507010), mouse IL-12β ELISA Kit (Abcam, ab236717), and mouse TNF-α ELISA Kit (Abcam, ab208348). The cytokines ELISA assays were performed according to manufacturer’s instructions provided by each ELISA kit. Data acquired from at least four individual experiments, and each assay was performed by three biological repetitions.

### Nitric oxide (NO) measurement

The accumulation of nitrite in the cell supernatant acted as an indicator of NO production. NO production could be estimated by determining nitrite concentration, measured with Griess reaction [38]. The Griess reagent included 0.1% *N*-(1-naphthyl)ethylenediamine dihydrochloride, 1% sulfanilamide, and 2.5% H_3_PO4. The monolayer cells were incubated with bacteria at an infection ratio 1:10 with 1 h. Then the supernatant of infected cells was harvested at 8 hpi. Equal volumes of Griess reagent and cell supernatant were mixed and incubated for 10 min at room temperature. A microplate reader (Biotek, USA) was used to determine the absorbance at 540 nm. Data acquired from at least four individual experiments, and each assay was performed by three biological repetitions. The nitrite concentration of cell supernatant was determined by sodium nitrite standard curve.

### Cells infection assays

Replication rates during wild-type FY26 and its variants were measured according to the described previously [40–42]. Briefly, the monolayer HD11 cells with three replicate wells were infected with each bacteria at a MOI of 10. After 1 h of infection, the bacteria in the cell supernatant were discarded, and the infected macrophages were incubated with gentamicin to kill the non-internal bacteria. After incubation with gentamicin at six-time points (2, 4, 6, 8, 10, and 16 hpi), the infected cells were washed with PBS and lysed using the 0.1% Triton X-100. The plate counting for the total number of internalized bacteria was carried out with the serially diluted cell suspension. The internalized bacteria at 2 hpi acted as the initial number of intracellular bacteria to determine the replication. Intracellular survival was measured by the bacterial number at different time points relative to initially internalized bacteria. These experiments were performed in triplicate with three biological repetitions.

### Immunofluorescent imaging assays

Immunofluorescent imaging assays were conducted to quantify the number of intracellular bacteria according to the previous description [30, 43]. Briefly, the monolayer HD11 cells were infected with wild-type FY26 and its variants at a MOI of 5. The infected cells at 4 hpi were washed twice with PBS, and fixed in 3% paraformaldehyde for 15 min. After incubation with 0.1% Triton X-100, the cells were treated with 5% BSA. Cells were incubated with a polyclonal rabbit anti-APEC O2:K1 serum, which was prepared with the APEC strain DE205B, its background (O2:K1; ST140; Phylogroup B2; isolated from duck), as the previous described [30, 33, 44]. Then the infected cells were treated with FITC goat anti-rabbit IgG (EarthOx, San Francisco, USA), DAPI, and Phalloidin (actin staining; TRITC conjugated) as the described above. Immunofluorescent imaging of bacteria was detected using a Zeiss LSM-510 META confocal laser scanning microscope. The number of bacteria within infected HD11 cells could be directly counted from immunofluorescent imaging. Data for quantification of the intracellular bacteria represented the average proliferation level of at least 100 infected cells. These experiments were performed in triplicate with three biological repetitions.

### Animal experiments

Ethics statement: All animal experimental protocols were handled according to the guidelines of Experimental Animal Management Measures of Jiangsu Province and were approved by the Laboratory Animal Monitoring Committee of Jiangsu Province, China.

The 7-day-old HBK-Q-SPF ducks for duck model were used to determine the effect of *acs*-*yjcH*-*actP* operon loss on APEC virulence as the previously described [45]. The duck groups (20 ducks for each group) were challenged intratracheally with bacteria at 5.0 × 10^5^ CFU/duck (dose/duck similar to LD_50_ of wild-type FY26). The mortality was calculated at the 7th day after post-infection, and the survival/mortality rates of eight groups were assessed. These experiments were performed in triplicate.

To measure the effect of *acs*-*yjcH*-*actP* operon loss on APEC colonization in vivo, the systemic infection experiment of duck model was conducted to assess the bacteria proliferation in duck lungs and blood [45]. The duck groups were challenged intratracheally with bacteria at 2.0 × 10^6^ CFU/duck (dose/duck similar to LD_90_ of wild-type FY26). 15 ducks for each group were euthanized and dissected at 24 hpi to conduct systemic infection experiment. The number of bacteria colonizing in duckling tissues (lungs, spleen, liver, brain, kidney) and level of bacteremia in the blood were determined as follows: organ samples and blood were obtained from infected ducks; samples were weighed, suspended in PBS (1 mL/g), and homogenized; the blood and homogenized tissues were serially diluted and plated on LB agar plates for counting.

The 10-day-old SPF chicks (QYH Biotech) for chick colisepticemia model were used to determine the effect of *acs*-*yjcH*-*actP* operon loss on APEC virulence as the previously described with the slightly modified system [22, 46]. To assess survival/mortality rates were challenged intratracheally with bacteria at 5.5 × 10^5^ CFU/chick (dose/chick similar to LD_50_ of wild-type FY26). For systemic infection experiment of chick colibacillosis model, the chick groups were challenged intratracheally with bacteria at 3.0 × 10^6^ CFU/chick (dose/chick similar to LD_90_ of wild-type FY26).

### Histological analyses

The left lungs of the control and APEC-infected ducks were excised, immediately fixed with formalin. The routine histology was performed, and duck lungs were embedded in paraffin. Sections (4–6 μm) were cut by rotary microtome and fixed on slides. Sample slides were deparaffinized in xylol, and then rehydrated in ethanol (100%, 96%, 80%, and 70%). Sections were stained with hematoxylin and eosin (HE). Pathological assessments were performed using a light microscope (Eclipse, Nikon, Tokyo, Japan).

### Bacterial cytotoxicity assay

To investigate the contribution of acetate assimilation system on APEC FY26 cytotoxicity in macrophages, cytotoxicity level was reflected through the lactate dehydrogenase (LDH) release test as previously described [43]. Cells with three replicate wells were infected with each bacteria at a MOI of 5, and cells culture supernatants after 2 hpi, 4 hpi, 8 hpi, and 12 hpi were collected and centrifuged to remove any suspended cells. CytoTox 96 nonradioactive cytotoxicity kit (Promega) was used to determine the LDH releases by HD11 cells according to the manufacturer’s instructions. The supernatant was incubated with substrate reagent for cytotoxicity kit at room temperature under the dark condition, and the absorbance of the mixture was measured at 490 nm. The spontaneous LDH release was measured by harvesting the supernatant from uninfected cells. Cytotoxicity level was counted as a percentage of the amount of LDH released from infected macrophages relative to that of uninfected cells, which were lysed with 10% Triton X-100, as follows: (sample LDH release − spontaneous LDH release)/(maximum LDH release − spontaneous LDH release) × 100%. Data acquired from at least four individual experiments, and each assay was performed by three biological repetitions.

## Results

### The host-induced transcription of APEC *actP*, *yjcH*, and *acs* genes during its infection in macrophages, not happened at non-phagocytic cells

**Figure 1:**
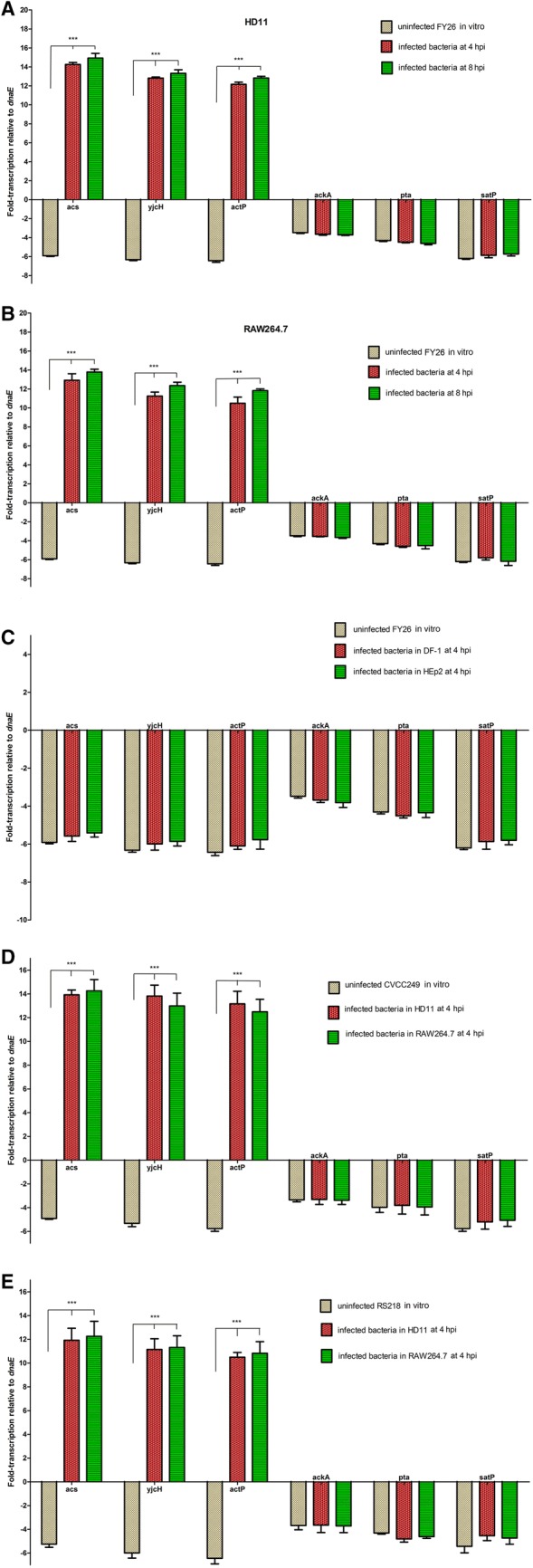
**The transcription levels of** ***acs***, ***yjcH***, ***actP***, ***ackA***, ***pta*, and** ***satP*** **genes in APEC/ExPEC strains.** qRT-PCR of these genes was determined relative to the transcription level of gene *dnaE*. Significant differences in qRT-PCR data were identified using one-way ANOVA statistical analysis (***P < 0.01). **A** The transcriptional differences of *acs*, *yjcH*, *actP*, *ackA*, *pta*, and *satP* genes during FY26 survival in HD11 macrophages relative to that in routine non-infectious condition at 41 °C in vitro. qRT-PCR data (uninfected FY26 in vitro; infected FY26 at 4 hpi or 8 hpi) acquired from three individual experiments were used to measure the differences (fold-change) of these genes transcription. **B** The transcriptional differences of *acs*, *yjcH*, *actP*, *ackA*, *pta*, and *satP* genes during FY26 survival in RAW264.7 macrophages relative to that in routine non-infectious condition at 37 °C in vitro. qRT-PCR data (uninfected FY26 in vitro; infected FY26 at 4 hpi or 8 hpi) was acquired from three individual experiments. **C** The transcription levels of *ackA*, *pta*, and *satP* genes between FY26 invasion in non-phagocytic cells (DF-1 or HEp2) relative to the cultured at 37 °C in vitro. qRT-PCR data (uninfected FY26 in vitro, infected FY26 in DF-1 cells at 4 hpi, and infected FY26 in HEp2 at 4 hpi) from three individual experiments were used to measure the differences (fold-change) of these genes transcription. **D** Transcriptional differences of *acs*, *yjcH*, *actP*, *ackA*, *pta*, and *satP* genes during strain CVCC249 survival in HD11 and RAW264.7 macrophages at 4 hpi relative to the routine condition in vitro. **E** Transcriptional differences of *acs*, *yjcH*, *actP*, *ackA*, *pta*, and *satP* genes during RS218 survival in HD11 and RAW264.7 macrophages at 4 hpi relative to the routine condition in vitro.

qRT-PCR was used to determine the transcription level of *acs*-*yjcH*-*actP* operon, *ackA*-*pta* operon, and *satP* gene in APEC/ExPEC strains (FY26, CVCC249, and RS218), which were the typical APEC/ExPEC dominant serotypes (O1:K1, O2:K1, and O18:K1) strains and belong to phylogroup B2 and ST95 [22, 27, 47]. qRT-PCR results showed that the transcription levels of *acs*, *yjcH*, *actP*, *ackA*, *pta*, and *satP* genes in APEC/ExPEC strains (FY26, CVCC249, and RS218) were close those of *dnaE* (*E. coli* housekeeping gene) under the routine culture condition at 41 °C in vitro (Figure 1). When wild-type FY26 was survived in HD11 macrophages, the transcription levels of *acs*, *yjcH*, and *actP* were obviously enhanced about 84.3-fold, 81.0-fold, and 78.2-fold at 4 hpi, respectively, compared to those under the routine culture condition (long logarithmic phase, LB broth for 6 h at 41 °C) (*P* < 0.01) (Figure 1A). The transcription levels of *acs*, *yjcH*, and *actP* in FY26 were also obviously enhanced at 8 hpi, respectively, relative to that for the cultured in vitro (*P* < 0.01) (Figure 1A). Additionally, the similar inducible up-regulation of *acs*, *yjcH*, and *actP* transcription could be detected when FY26 infected RAW264.7 at 37 °C (Figure 1B). However, there were no obvious differences in the transcription of *ackA*, *pta*, and *satP* genes between FY26 invasion in non-phagocytic cells (DF-1 or HEp2) and the cultured at 37 °C in vitro (*P* ≥ 0.05) (Figure 1C). Similarly, during CVCC249 survival in HD11 macrophages, the transcription levels of *acs*, *yjcH*, and *actP* were obviously enhanced about 68.4–fold, 73.5-fold, and 75.9-fold at 4 hpi, respectively, compared to those under the routine culture condition at 41 °C (*P* < 0.01) (Figure 1D). Similarly, the inducible up-regulation of *acs*, *yjcH*, and *actP* transcription could be detected when CVCC249 infected RAW264.7 macrophages at 37 °C, and the transcription levels of *acs*, *yjcH*, and *actP* in CVCC249 were enhanced about 70.1-fold, 69.1-fold, and 72.0-fold at 4 hpi, respectively, relative to those from in vitro culture (*P* < 0.01) (Figure 1D). Moreover, during RS218 survival in macrophages, the transcription levels of *acs*, *yjcH*, and *actP* were also obviously enhanced when compared with those of the routine culture condition (*P* < 0.01) (Figure 1E). However, there was no obvious difference for the transcription of *ackA*, *pta*, and *satP* genes between APEC survival in HD11 macrophages and the in vitro cultured (*P* ≥ 0.05).

These results indicated that the transcription of *actP*, *yjcH*, and *acs* genes presented host-induced trait during APEC infection in macrophages, but not in non-phagocytic cells. This characteristic suggested that the acetate assimilation system encoded by the *acs*-*yjcH*-*actP* operon might play roles in APEC survival and replication within macrophages.

### Acetate assimilation system encoded by *acs*-*yjcH*-*actP* operon was essential for APEC growth under acetic acid as the sole carbon source

**Figure 2:**
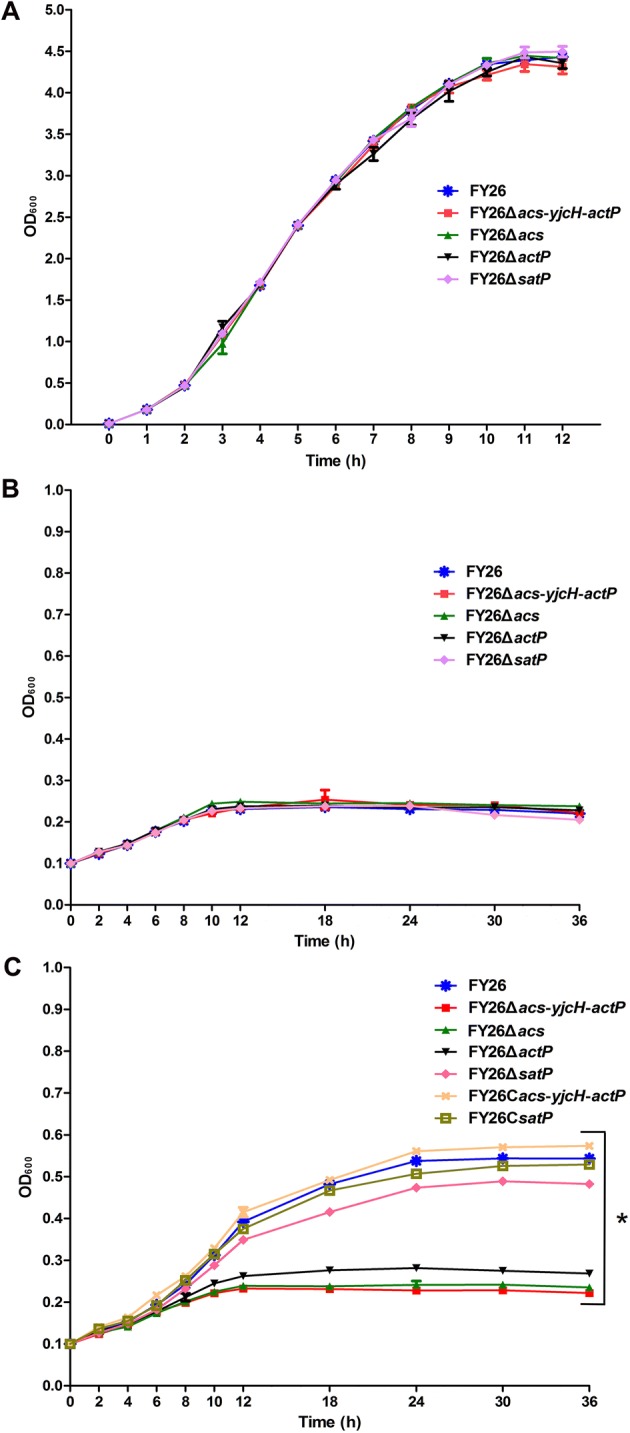
**Acetate assimilation system encoded by** ***acs*-*yjcH*-*actP*** **operon contributed to APEC growth. A** Growth profiles of APEC wild-type FY26, mutants FY26Δ*acs*-*yjcH*-*actP*, FY26Δ*acs*, FY26Δ*actP*, and FY26Δ*satP* under the LB culture (rich media) condition. **B** Growth profiles of APEC wild-type FY26, mutants FY26Δ*acs*-*yjcH*-*actP*, FY26Δ*acs*, FY26Δ*actP*, and FY26Δ*satP* in M9 minimal medium without acetic acid. **C** Growth profiles of APEC wild-type FY26, mutants and the corresponding complemented strains (FY26C*acs*-*yjcH*-*actP* and FY26C*satP*) under M9 minimal medium plus acetic acid (60 mM, 0.36%) condition. The two-way ANOVA was performed for growth curves (*P < 0.01).

To clarify the roles of this acetate assimilation system during APEC infection, the whole *acs*-*yjcH*-*actP* operon was deleted in wild-type FY26, and the single deletion mutants of *acs*, *actP* or *satP* gene were also constructed on strain FY26 (Additional file 1). The complemented strains FY26C*acs*-*yjcH*-*actP* and FY26C*satP* were generated by transformation of plasmids pSTV28-*acs*-*yjcH*-*actP* and pSTV28-*satP* to the corresponding mutants, respectively. Growth in vitro was first evaluated in a series of analyses. As showed in Figure 2A, the growth curves showed that deletion of *acs*-*yjcH*-*actP* operon or *satP* gene in strain FY26 had no effect on *E. coli* growth under the LB cultured (rich media) condition. Similarly, there was no difference among the growth curves of wild-type FY26, mutants FY26Δ*acs*-*yjcH*-*actP*, FY26Δ*acs*, FY26Δ*actP*, and FY26Δ*satP* in M9 minimal medium without acetic acid (Figure 2B). For M9 medium plus acetic acid (60 mM, 0.36%), our result showed that *satP* mutant displayed a slight lag phase compared with the growth curve of wild-type FY26. However, the deletion of *acs*-*yjcH*-*actP* operon in APEC FY26 have a significant alteration in growth curves under M9 medium plus acetic acid condition. This result suggested that acetate assimilation system encoded by *acs*-*yjcH*-*actP* operon was essential for bacterial growth when acetic acid acted as the sole carbon and energy source (Figure 2C). FY26Δ*acs* and FY26Δ*acs*-*yjcH*-*actP* grew equally in minimal medium (M9 plus 60 mM acetic acid) and display a longer lag phase relative to the growth curve of mutant FY26Δ*actP*. Moreover, the growth of the complemented strains FY26C*acs*-*yjcH*-*actP* and FY26C*satP* was restored to the level of wild-type FY26 (Figure 2C).

### Acetate assimilation system encoded by *acs*-*yjcH*-*actP* operon was essential for APEC intracellular replication within macrophages

**Figure 3:**
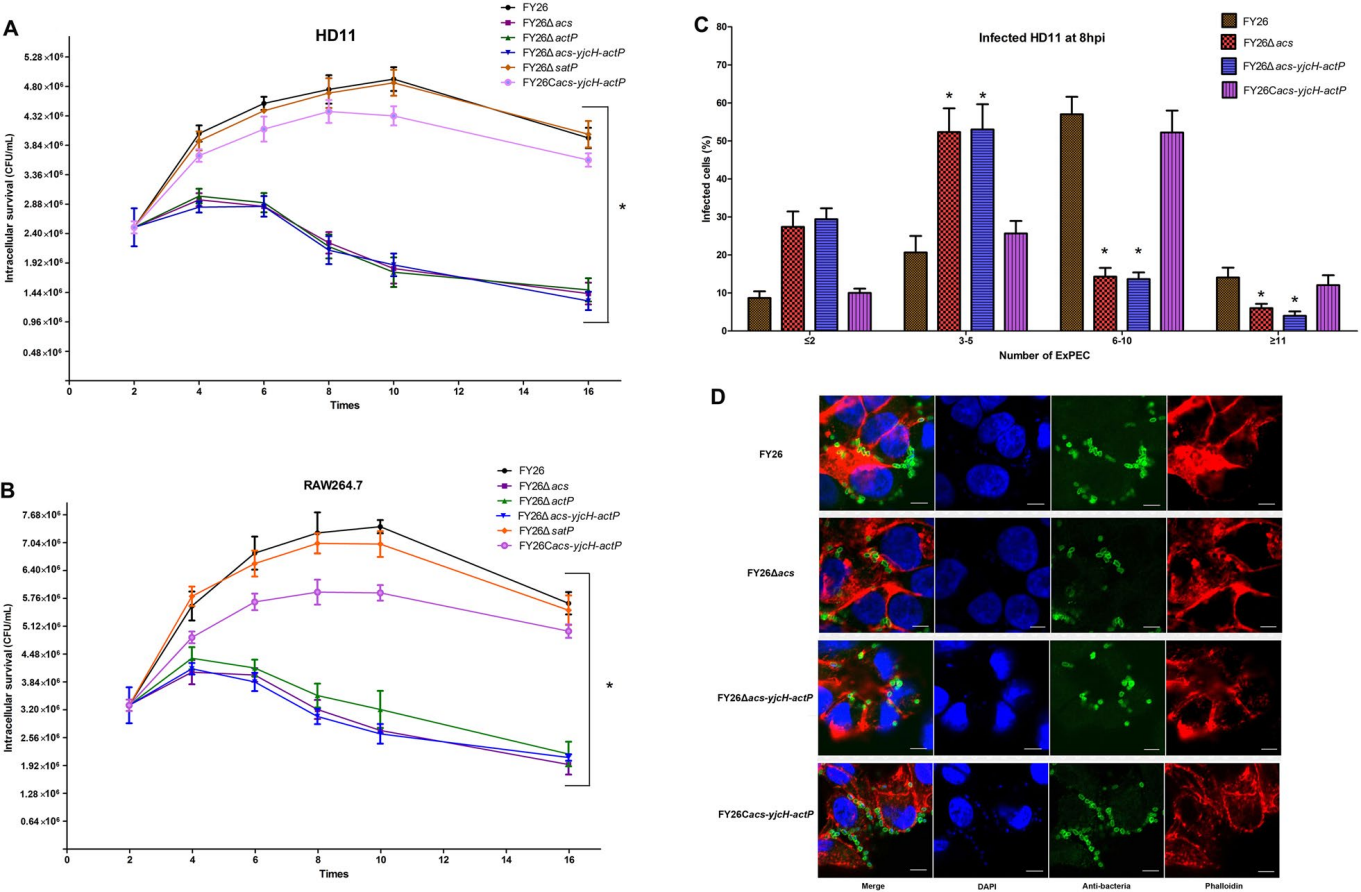
**Acetate assimilation system encoded by** ***acs*-*yjcH*-*actP*** **operon contributed to APEC intracellular replication within macrophages. A** Deletion of *acs*-*yjcH*-*actP* operon impaired FY26 intracellular replication within HD11 macrophages. The survival rates of wild-type FY26, four mutants (FY26Δ*acs*, FY26Δ*actP*, FY26Δ*acs*-*yjcH*-*actP*, and FY26Δ*satP*), and the complemented strain FY26C*acs*-*yjcH*-*actP* were measured to investigate the contribution of the acetate assimilation system on APEC proliferation. **B** To determine the contribution of acetate assimilation system on APEC replication within RAW264.7 macrophages. Macrophages were infected at a MOI of 10. Data acquired from at least four individual experiments, and each assay was performed by three biological repetitions. The significant differences were identified using two-way ANOVA analysis (*P < 0.01), and the mean values ± SEs were shown in each plotting. **C**, **D** Determine the effect of acetate assimilation system on APEC replication within macrophages by immunofluorescence assays. HD11 cells were infected at a MOI of 5. **C** Quantification of wild-type FY26, FY26Δ*acs*, FY26Δ*acs*-*yjcH*-*actP*, and FY26C*acs*-*yjcH*-*actP* intracellular proliferation in macrophages was indicated. The percentage of infected HD11 cells containing ≤ 2, 3 to 5, 6 to 10, and ≥ 11 bacteria per cell at 8 hpi was counted by fluorescence microscopy. Data for quantification of bacteria proliferation was acquired from more than 100 infected cells for three independent experiments. The significant differences were identified using one-way ANOVA analysis (*P < 0.01), and the mean values ± SEs were shown. **D** Representative confocal microscopy images of wild-type FY26, FY26Δ*acs*, FY26Δ*acs*-*yjcH*-*actP*, and FY26C*acs*-*yjcH*-*actP* intracellular proliferation in macrophages at 8 hpi were shown. The infected HD11 cells were fixed and labeled with DAPI and Phalloidin (actin staining; TRITC conjugated). Bacteria were incubated with an anti-APEC antibody and immediately labeled with a fluorescent secondary antibody (Alexa 488, green). Scale bar = 10 μm.

Previous studies have revealed that APEC can survive and replicate within macrophages [13, 40, 48]. However, the metabolic strategies utilized by intracellular APEC to enhance proliferative capacity in host phagocytosis cells remain poorly understood. Acetate can act as a carbon source to support energy homeostasis in prokaryotic or eukaryotic cells. As expected, the replication ability of the mutants (lacking *acs*, *actP,* or the whole operon) survival in HD11 macrophages was significantly impaired relative to that of wild-type FY26. As shown in Figure 3A, the survival rate of the FY26Δ*acs* at 4 hpi and 8 hpi were decreased to 72.5% and 46.2% relative to those of wild-type FY26, respectively (*P* < 0.01). Similarly, the survival rate of the FY26Δ*actP* at 4 hpi and 8 hpi were just only about 74.1% and 44.9% compared to that of wild-type FY26, respectively (*P* < 0.01). Additionally, consistent with the single deletion of *acs* or *actP* gene in FY26, the results showed that deletion of *acs*-*yjcH*-*actP* operon impaired the APEC replication within HD11 macrophages (*P* < 0.01). The survival of the complemented strain FY26C*acs*-*yjcH*-*actP* was restored to the level of wild-type FY26. However, there was no obvious difference between FY26Δ*satP* and wild-type FY26 (*P* ≥ 0.05). As shown in Figure 3B, the replication ability of these mutants (lacking *acs*, *actP,* or the whole operon) survival in RAW264.7 macrophages was also significantly impaired compared with that of wild-type FY26 (*P* < 0.01).

Immunofluorescence assay was performed to determine whether *acs*-*yjcH*-*actP* operon loss affected the APEC survival or replication within HD11 macrophages. At a MOI of 5, the presence of bacteria can be detected in more than 96% of infected cells at the initial time point (2 hpi). Numbers of bacteria per infected HD11 cells at different time points were divided into the following categories: ≤ 2, 3 to 5, 6 to 10, and ≥ 11. The number of bacteria (FY26, FY26Δ*acs*-*yjcH*-*actP*, and FY26C*acs*-*yjcH*-*actP*) in 100 infected cells at 2 hpi and 4 hpi was counted. As a result, intracellular bacteria were generally less than 5, and the statistical differences were not observed by immunofluorescence at early time points. As shown in Figure 3C, the majority (~71.1%) of the HD11 macrophages infected with wild-type FY26 contained ≥ 6 bacteria at 8 hpi, and the percentage of infected cells containing 6 to 10 bacteria was ~57%. In contrast, the percentage of FY26Δ*acs*-*yjcH*-*actP* infected macrophages containing ≥ 6 bacteria decreased significantly to approximately ~20.3% at 8 hpi. Bacteria counting of FY26Δ*acs* infected macrophages was similar to the result of FY26Δ*acs*-*yjcH*-*actP* (Figure 3C). Furthermore, bacteria counting revealed that the replication ability of the complemented FY26C*acs*-*yjcH*-*actP* was restored to the similar level of wild-type FY26. Representative fluorescence microscopy images of the strains replication within HD11 macrophages at 8 hpi were shown in Figure 3D. These results clarified that acetate assimilation system encoded by *acs*-*yjcH*-*actP* operon acted as an intracellular survival factor, and the acetate metabolic requirement of APEC promoted its intracellular proliferation within macrophages.

### The acetate assimilation system was required for APEC inducing macrophage injury

**Figure 4:**
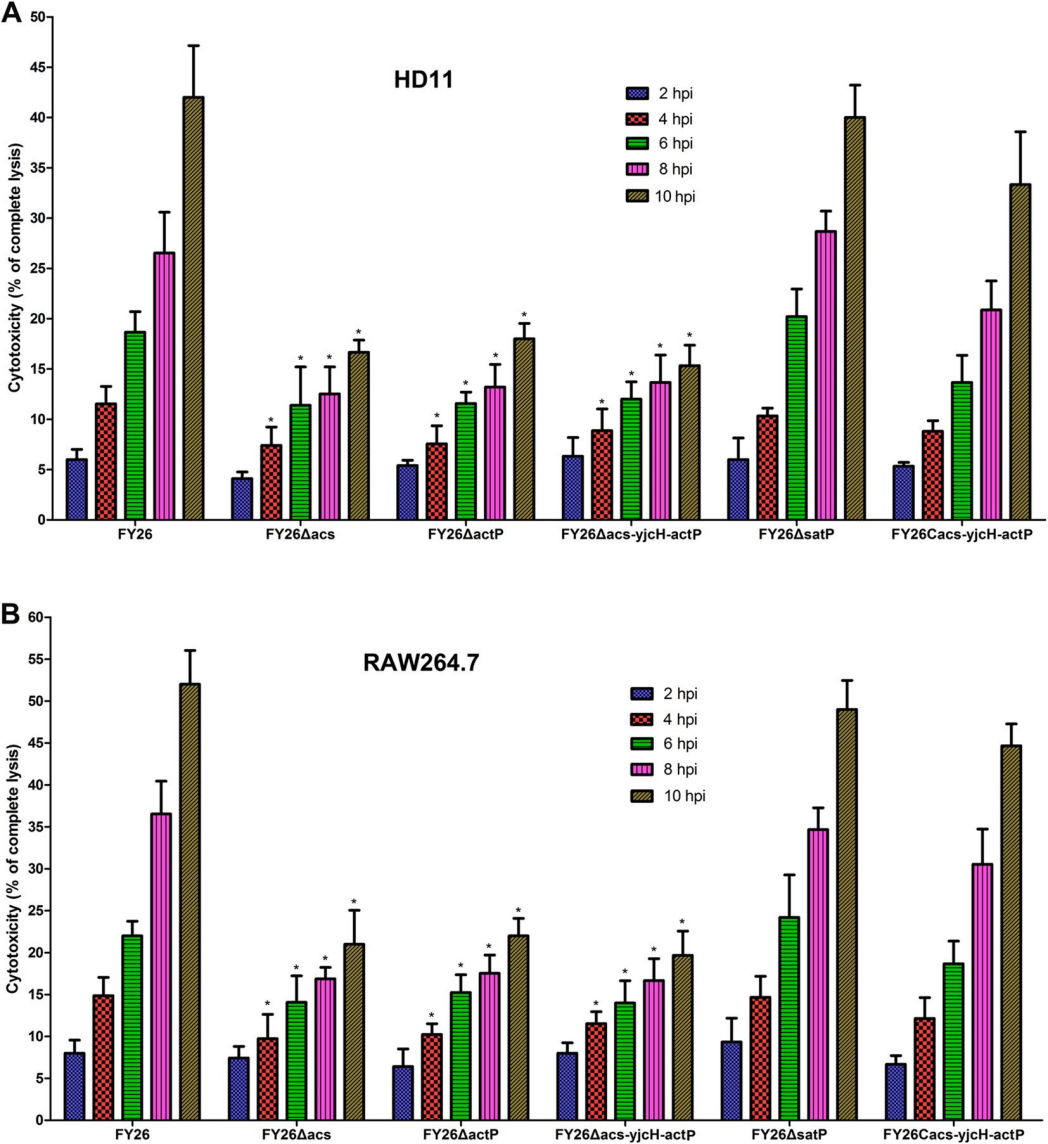
**Acetate assimilation system encoded by** ***acs*-*yjcH*-*actP*** **operon was required for APEC-induced cytotoxicity to macrophages. A** To investigate the cytotoxic effect of *acs*, *actP* or *satP* mutants on HD11 macrophages. Cells culture supernatant after 2 hpi, 4 hpi, 8 hpi, and 12 hpi was collected to determine the LDH releases by HD11 cells. Cytotoxicity level was counted as a percentage of the amount of LDH released from infected macrophages relative to that of uninfected cells lysed with 10% Triton X-100. **B** To determine the cytotoxic effect of *acs*, *actP* or *satP* mutants on RAW264.7 macrophages. Data acquired from at least four independent experiments performed in triplicate, and the mean values ± the standard errors were shown. Statistical differences were determined using two-way ANOVA analysis (*P < 0.01).

Besides being a fuel for central metabolism, the emerging studies indicate the functional role of the acetate metabolism in host central energy homeostasis and critical correlation with multiple host physiological features [17, 49]. In independent experiments, we assessed the effect of acetate assimilation system on host cell damage during APEC interaction with macrophages. The cytotoxic effect of *acs*, *actP* or *satP* mutants to macrophages (HD11 and RAW264.7) was determined by quantifying the amount of the lactate dehydrogenase (LDH) released by the infected macrophages. LDH released from infected HD11 macrophages was detected at four time points (2 hpi, 4 hpi, 8 hpi, and 12 hpi). The data showed that the amount of LDH release was gradually increased during FY26 interaction with HD11 cells, whereas macrophages exposed to the *acs*, *actP*, or *acs*-*yjcH*-*actP* mutant exhibited decreased LDH release level relative to that of wild-type FY26 (*P* < 0.01) (Figure 4A). Furthermore, the LDH testing results revealed that the cytotoxicity level exposed to complemented FY26C*acs*-*yjcH*-*actP* was restored to the similar level of wild-type FY26. The deletion of *satP* gene in FY26 had no obvious effect on cytotoxicity in HD11 macrophages (*P* > 0.05). Moreover, the LDH testing results demonstrated that the cytotoxic levels during RAW264.7 macrophages exposed to the *acs*, *actP*, and *acs*-*yjcH*-*actP* mutants were significantly lower relative to that of wild-type FY26 (*P* < 0.01) (Figure 4B). Similarly, the cytotoxicity on RAW264.7 exposed to the complemented FY26C*acs*-*yjcH*-*actP* was restored to the similar level of the wild-type FY26 (*P* > 0.05). The deletion of *satP* gene in FY26 had no obvious effects on cytotoxicity to RAW264.7 macrophages (*P* > 0.05). Mitochondrion toxicity was also conducted in this study, but there were no differences on mitochondrion toxicity for macrophages exposed to between wild-type FY26 and mutants (data not shown). Taken together, these data indicated that the acetate assimilation system was required for APEC inducing macrophage injury during its intracellular replication.

### Over-expression of APEC acetate assimilation system enhanced the production of nitric oxide (NO) and proinflammatory cytokines in infected macrophages

**Figure 5:**
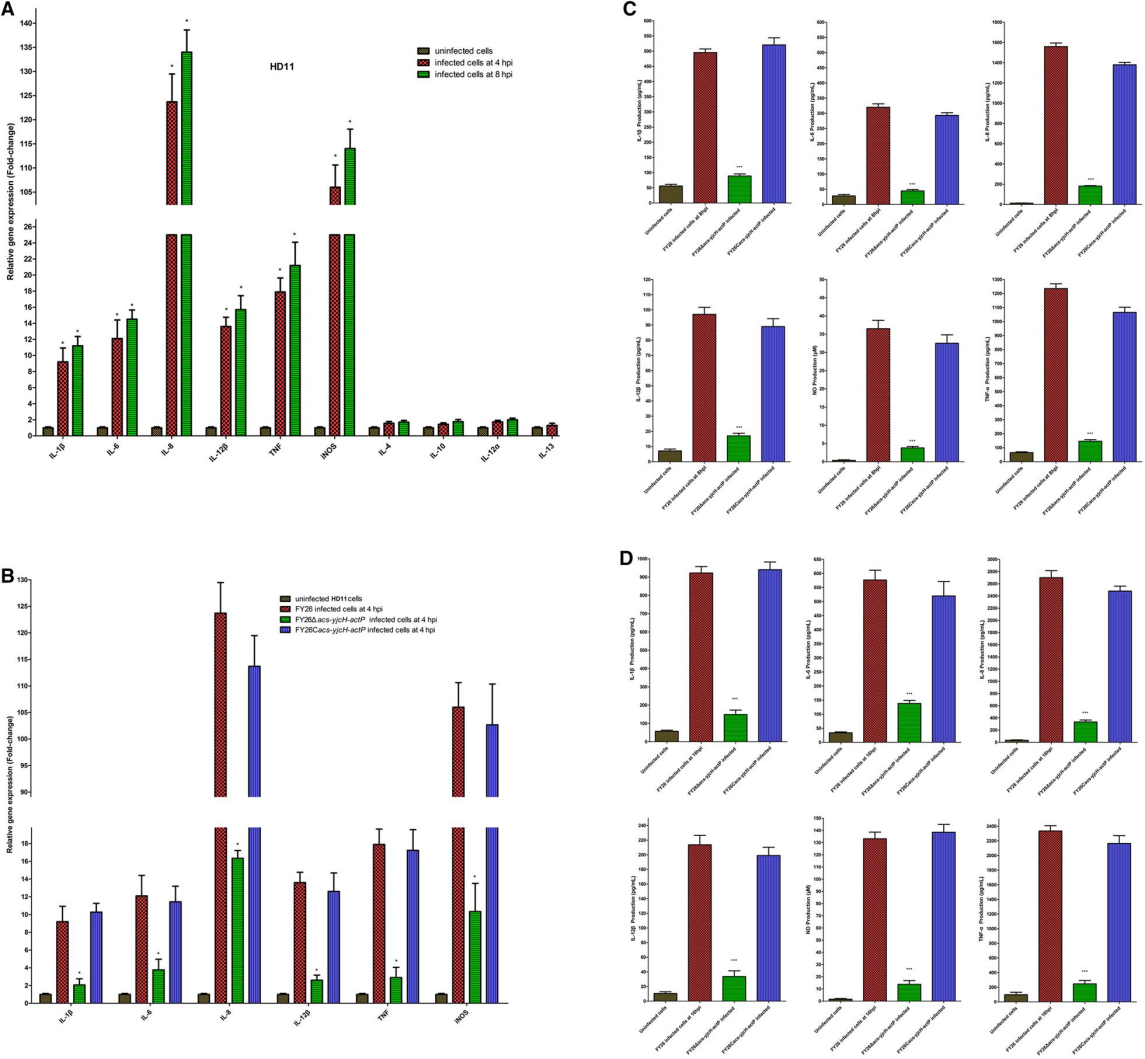
**The over-expression of acetate assimilation system promoted pro-inflammatory responses of APEC infected macrophages. A** Determining the transcriptional differences of pro-inflammatory cytokines (IL-1β, IL-6, IL-8, IL-12β, and TNF-α), anti-inflammatory cytokines (IL-4, IL-10, and IL-13), and iNOS between FY26 infected HD11 cells and non-infectious cells. qRT-PCR data acquired from three independent experiments were used to determine the transcription of selected cytokines genes during wild-type FY26 replication within HD11 cells at 4 hpi and 8 hpi. Significant differences were statistically determined using one-way ANOVA analysis (*P < 0.01), and the mean values ± the standard errors were shown. **B** Determining the effect of *acs*-*yjcH*-*actP* deletion on proinflammatory responses in RAW264.7 macrophages. Transcriptional differences of IL-1β, IL-6, IL-8, IL-12β, TNF-α, and iNOS among wild-type FY26, FY26Δ*acs*-*yjcH*-*actP*, and FY26C*acs*-*yjcH*-*actP* infected HD11 cells at 4 hpi were analyzed by qRT-PCR relative to that of uninfected HD11 cells. **C** Expression analysis of nitric oxide (NO) and secreted cytokines in FY26, FY26Δ*acs*-*yjcH*-*actP*, and FY26C*acs*-*yjcH*-*actP* infected RAW264.7 macrophages at 8 hpi. The cultured supernatant of infected cells was harvested at 8 hpi or the uninfected cultured. ELISA assay was conducted to evaluate the concentration of NO and proinflammatory cytokines (IL-1β, IL-6, IL-8, IL-12β, and TNF-α) in the culture supernatant of RAW264.7 macrophages to determine the effect of *acs*-*yjcH*-*actP* deletion on proinflammatory responses in RAW264.7 macrophages. **D** Expression analysis of nitric oxide (NO) and secreted cytokines in FY26, FY26Δ*acs*-*yjcH*-*actP*, and FY26C*acs*-*yjcH*-*actP* infected RAW264.7 macrophages at 16 hpi. Statistical difference was determined using one-way ANOVA analysis (*, *P* < 0.01), and the mean values ± the standard errors were shown.

Once infected by a pathogen, macrophages can secrete large amounts of pro-inflammatory and anti-inflammatory cytokines and interleukins, and enhance the production of nitric oxide (NO). An important incentive for bacteria successful survival and proliferation within infected macrophages is the excessive inflammation due to the imbalance of pro-inflammatory and anti-inflammatory responses, accompanied by immunity failure and body inflammatory damage [4]. To investigate whether the over-expression of acetate assimilation system could induce inflammatory responses of APEC infected macrophages, we determined the cytokine responses between wild-type FY26 and FY26Δ*acs*-*yjcH*-*actP* infected HD11 macrophages at 4 hpi by qRT-PCR. Referring to recent research [15, 37, 39, 50], the gene transcription involved in pro-inflammatory interleukins (IL-1β, IL-6, IL-8, IL-12α, and IL-12β), tumor necrosis factor-α (TNF-α), inducible nitric oxide synthase (iNOS), and anti-inflammatory cytokines (IL-4, IL-10, and IL-13) was examined. As shown in Figure 5A, the transcriptional levels of these targeted genes encoding pro-inflammatory cytokines (IL-1β, IL-6, IL-8, IL-12β, and TNF-α) and iNOS were significantly up-regulated in FY26 infected HD11 at 4 hpi and 8 hpi relative to that of the control (uninfected HD11 cultures), especially the excessive expression of IL-8, TNF, and iNOS (*P* < 0.01). Unlike the pro-inflammatory cytokines, the transcription of anti-inflammatory cytokines (IL-4, IL-10, and IL-13) presented a lower level in infected HD11, which were similar to that of the uninfected HD11 cultures (*P* > 0.05) (Figure 5A). This result indicated that infected HD11 macrophages presented excessively pro-inflammatory responses. However, deletion of *acs*-*yjcH*-*actP* genes in FY26 had obvious effects on the transcription of the pro-inflammatory cytokines in infected HD11 macrophages. qRT-PCR results showed that the transcription of IL-1β, IL-6, IL-8, IL-12β, TNF-α, and iNOS was obviously down-regulated in FY26Δ*acs*-*yjcH*-*actP* infected macrophages compared to that in wild-type FY26 infected cultures at 4 hpi (*P* < 0.01) (Figure 5B). Similarly, the transcription of the pro-inflammatory cytokines in FY26C*acs*-*yjcH*-*actP* infected macrophages was restored to the similar level for the wild-type FY26 infected condition (*P* > 0.05).

To determine the effect of *acs*-*yjcH*-*actP* deletion on proinflammatory responses in RAW264.7 macrophages, ELISA assay was conducted to evaluate the production of nitric oxide (NO) and proinflammatory cytokines (IL-1β, IL-6, IL-8, IL-12β, and TNF-α) in RAW264.7 macrophages. As shown in Figure 5C, the release levels of NO, IL-1β, IL-6, IL-8, IL-12β, and TNF-α for FY26Δ*acs*-*yjcH*-*actP* infected RAW264.7 macrophages were significantly lower than that of wild-type FY26 infected cultures at 8 hpi (*P* < 0.01). The release levels of NO, IL-1β, IL-6, IL-8, IL-12β, and TNF-α in FY26C*acs*-*yjcH*-*actP* infected RAW264.7 macrophages were partially restored to the levels for the wild-type FY26 infected cell cultures (*P* > 0.05). Moreover, the cytokine production at a later time point (16 hpi) was measured. As shown in Figure 5D, the release levels of NO, IL-1β, IL-6, IL-8, IL-12β, and TNF-α for FY26Δ*acs*-*yjcH*-*actP* infected RAW264.7 macrophages were also decreased relative to that of wild-type FY26 infected cultures at 16 hpi (*P* < 0.01). In this section, the results showed that the over-expression of APEC acetate assimilation system promoted excessively pro-inflammatory responses through enhancing the production of nitric oxide (NO) and proinflammatory cytokines in infected macrophages. The recent research shows that the acetate plays a critical role in the modulation of host inflammatory response [17, 49, 51]. Therefore, the host acetate consumption during APEC survival in macrophages might cause host immunomodulatory disorders and excessively pro-inflammatory responses.

### The acetate assimilation system was increasingly expressed during APEC early colonization in lung tissues in vivo for duck model

**Figure 6:**
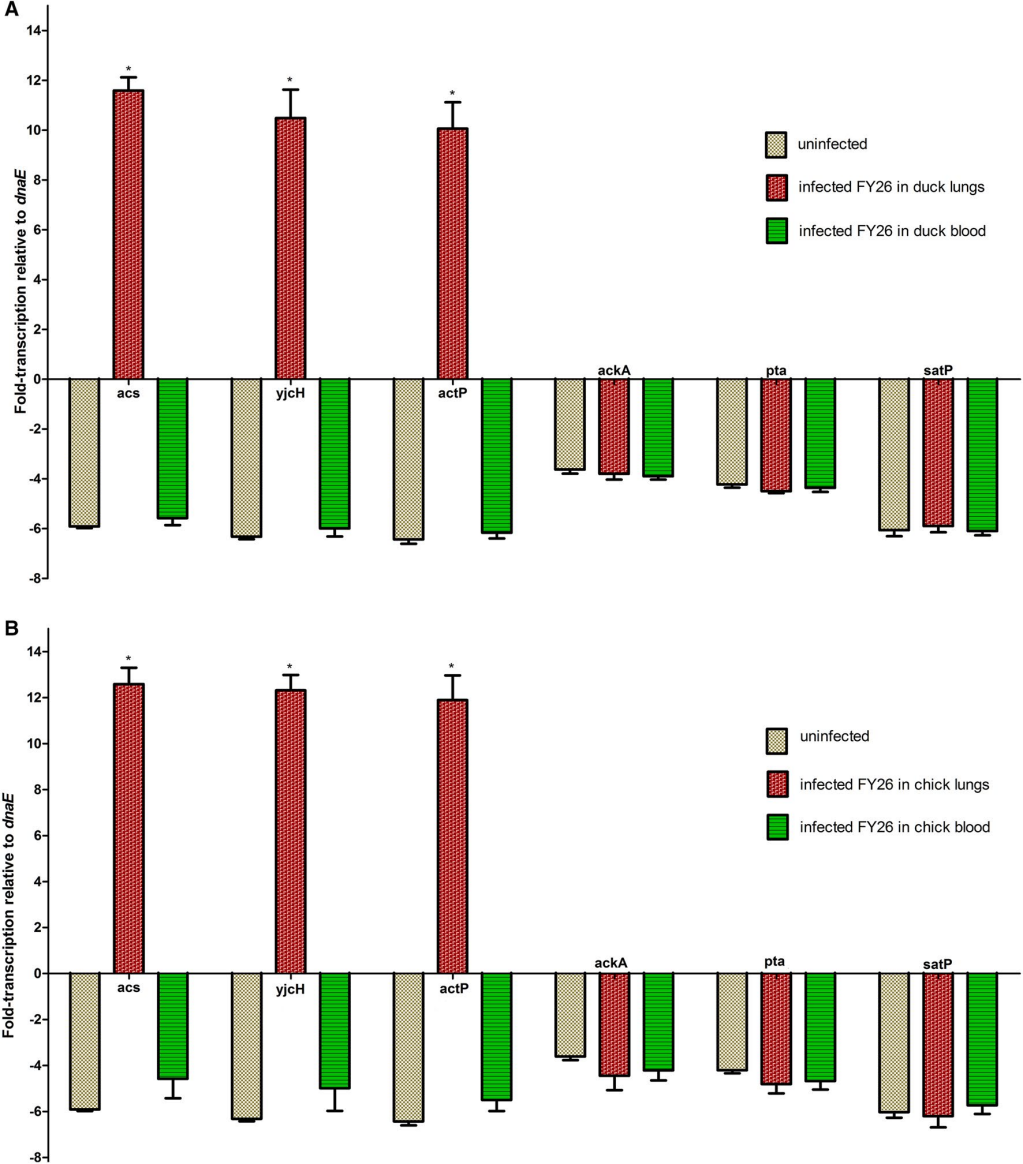
**The expression of acetate assimilation system encoded by** ***acs*-*yjcH*-*actP*** **operon was highly induced during APEC early colonization in lung tissues in vivo. A** For duck model, qRT-PCR data acquired from three individual experiments were used to determine the transcription of *acs*, *yjcH*, *actP*, *satP*, *ackA*, and *pta* genes during wild-type FY26 colonization in duck organs in vivo at 24 hpi. Transcriptional differences of these genes were determined relative to that of the routine condition in vitro. **B** For chick model, transcriptional differences of *acs*, *yjcH*, *actP*, *satP*, *ackA*, and *pta* genes during FY26 colonization in chick tissues in vivo at 24 hpi were determined relative to that of the routine condition in vitro. Significant differences of qRT-PCR results were identified using one-way ANOVA statistical analysis (*P < 0.01).

To further determine whether the acetate assimilation system encoded by *acs*-*yjcH*-*actP* operon affected the virulence of wild-type FY26, we detected the expression of acetate assimilation system during FY26 colonization in vivo in avian colisepticemia model. The duck is used as a typical avian infection model to evaluate the virulence of APEC isolates. The 7-day-old ducklings were inoculated intratracheally with bacteria at 2.0 × 10^6^ CFU, and qRT-PCR was used to determine the transcription of *acs*, *yjcH*, *actP*, and *satP* genes during FY26 colonization in duck organs (lung, liver and spleen) and proliferation in blood in vivo at 24 hpi. As shown in Figure 6A, our result indicated that the transcription levels of *acs*, *yjcH*, and *actP* were up-regulated by 68.4-fold, 66.3-fold, and 64.3–fold during FY26 colonization in duck lung tissues compared with that from the routine culture in vitro (*P* < 0.01) (Figure 6A). However, the transcription levels of *acs*, *yjcH*, *actP*, and *satP* genes for wild-type FY26 isolated from bacteremia in vivo were close to that from the routine culture in vitro (*P* > 0.05) (Figure 6A). Moreover, the transcription levels of these genes during wild-type FY26 colonization in duck livers or spleen were also close to that of *dnaE*, and didn’t present induced up-regulation (data not shown). Similar to the results of duck infection model, the transcription levels of *acs*, *yjcH*, and *actP* were obviously up-regulated during FY26 colonization in chick lung tissues compared with those from the routine culture (*P* < 0.01) (Figure 6B).

### Deletion of *actP*/*yjcH*/*acs* genes attenuated APEC virulence and colonization capability in avian lungs in vivo for avian infection models

**Figure 7:**
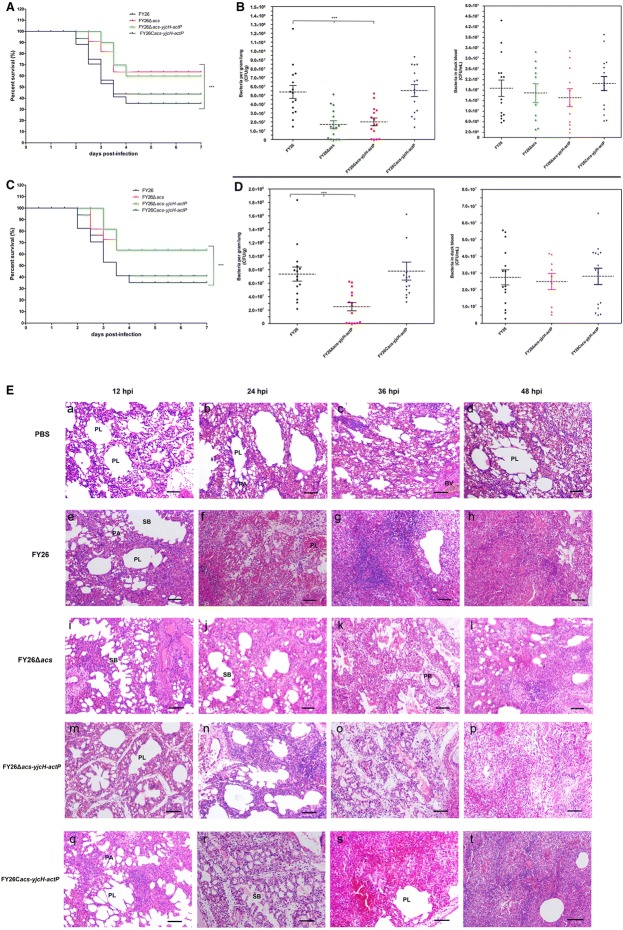
**The acetate assimilation system encoded by** ***acs*-*yjcH*-*actP*** **operon contributed to APEC virulence and phenotype fitness. A** The mortality rates of wild-type FY26 and variants were determined by duck model to investigate the effect of *acs*-*yjcH*-*actP* deletion on the virulence of APEC. Survival rate was measured after 7 days post-infection. The two-way ANOVA was performed for survival assays (*P < 0.01). **B** Determining the effect of *acs*-*yjcH*-*actP* operon deletion on APEC colonization in vivo. Systemic infection experiment of the duck infection model was performed to assess the bacteria proliferation in duckling organs and blood at 24 hpi. Statistical significances were identified using a nonparametric Mann–Whitney U test (*P < 0.01). **C** The mortality rates of wild-type FY26, the mutant FY26Δ*acs*-*yjcH*-*actP*, and the complemented FY26C*acs*-*yjcH*-*actP* were determined by chick model. **D** Systemic infection experiment of the duck infection model was performed to assess the bacteria proliferation in chick organs and blood at 24 hpi. **E** Histopathology of lungs from ducks infected intratracheally with wild-type FY26, FY26Δ*acs*, FY26Δ*acs*-*yjcH*-*actP*, FY26C*acs*-*yjcH*-*actP*, and the negative control PBS. Duck lungs at 12 hpi, 24 hpi, 36 hpi, or 48 hpi were fixed, and sections were stained with HE. The pathological changes of the lungs were observed under a light microscope. a–d The image observation of lung sections from PBS-inoculated ducks at 12 hpi, 24 hpi, 36 hpi, or 48 hpi, respectively. e–h The lesions of lung sections from FY26-infected ducks at four time points. i–l The lesions of lung sections from FY26Δ*acs*-infected ducks. m–p The lesions of lung sections from FY26Δ*acs*-*yjcH*-*actP*-infected ducks. q–t The lesions of lung sections from FY26C*acs*-*yjcH*-*actP*-infected ducks. Scale bars 100 μm. BV, blood vessel; PB, primary bronchus; SB, secondary bronchus; PL, parabronchial lumen; PA, pulmonary alveolus.

To investigate the effect of *acs*-*yjcH*-*actP* deletion on the virulence of APEC strain FY26, the mortality rates of wild-type FY26, FY26Δ*acs*, FY26Δ*acs*-*yjcH*-*actP*, and the complemented FY26C*acs*-*yjcH*-*actP* was determined in the duck model. Each duckling was challenged intratracheally with bacteria at 5.0 × 10^5^ CFU. As shown in Figure 7A, the survival curves indicated that FY26Δ*acs* and FY26Δ*acs*-*yjcH*-*actP* decreased the mortality rates and postponed the death peak of the infected ducks compared with that of wild-type FY26 (*P* < 0.01). Moreover, the mortality rate of the complemented FY26C*acs*-*yjcH*-*actP* was restored to the similar level of wild-type FY26 (*P* > 0.05). However, the *acs*-*yjcH*-*actP* operon deletion had no effect on the APEC virulence in the murine sepsis model (data not shown). The result indicated that the acetate assimilation system conferred a fitness advantage of APEC infection in duck model relative to that of the murine sepsis model.

To measure the effect of *acs*-*yjcH*-*actP* operon deletion on APEC colonization in vivo, the systemic infection experiment of the duck model was performed to assess the bacteria proliferation in duckling organs and septicemia level. As shown in Figure 7B, the early colonization capacity in duckling lungs among the four strains was determined at 24 hpi. The mutants FY26Δ*acs* and FY26Δ*acs*-*yjcH*-*actP* exhibited decreased colonization capacity in duckling lungs relative to that of wild-type FY26 and the complemented FY26C*acs*-*yjcH*-*actP* (*P* < 0.01). About 65% of the ducks infected with the mutants FY26Δ*acs* and FY26Δ*acs*-*yjcH*-*actP* presented bacteremia symptoms compared to the ducks challenged by wild-type FY26, suggesting that the loss of *acs*-*yjcH*-*actP* operon might impair APEC capability to pass through the immune defense of duck lung tissues into the bloodstream (*P* < 0.01). However, the septicemia for the alive ducklings infected with the mutants was not detected. For the ducklings presenting bacteremia symptoms, the statistics of bacteremia levels showed that bacteria proliferation in the blood for FY26Δ*acs* and FY26Δ*acs*-*yjcH*-*actP* infected ducks had no significant difference with that of wild-type FY26 and the complemented FY26C*acs*-*yjcH*-*actP* (Figure 7B) (*P* > 0.05), suggesting acetate assimilation system encoded by *acs*-*yjcH*-*actP* operon was not involved in bacteria proliferation in blood in vivo. Moreover, there was no significant difference for the bacteria proliferation/colonization of these strains among duckling spleen, liver, brain, and kidney tissues (data not shown). The results of systemic infection experiment illustrated that the acetate assimilation system conferred an advantage of the APEC early colonization in duck respiratory system in vivo.

Similar to the results of duck infection model, deletion of *actP*/*yjcH*/*acs* genes attenuated APEC virulence and colonization capability in avian lungs in vivo for chicken infection model. As shown in Figure 7C, the survival curves indicated that FY26Δ*acs*-*yjcH*-*actP* decreased the mortality rate and postponed the death peak of the infected chicks compared with that of wild-type FY26 (*P* < 0.01). The mutant FY26Δ*acs*-*yjcH*-*actP* exhibited decreased colonization capacity in chick lungs relative to that of wild-type FY26 and the complemented FY26C*acs*-*yjcH*-*actP* (*P* < 0.01) (Figure 7D). Similar to the duck model, 60% of the chicks infected with the mutant FY26Δ*acs*-*yjcH*-*actP* presented bacteremia symptoms compared to the ducks challenged by wild-type FY26. For the chicks presenting bacteremia symptoms, the statistics of bacteremia levels showed that bacteria proliferation in the blood for FY26Δ*acs* and FY26Δ*acs*-*yjcH*-*actP* infected ducks had no significant difference with that of wild-type FY26 and the complemented FY26C*acs*-*yjcH*-*actP* (Figure 7D).

We conducted histopathological analysis of the duck lungs at 12 hpi, 24 hpi, 36 hpi, and 48 hpi after duckling challenged intratracheally with four strains (wild-type FY26, the mutants FY26Δ*acs*, FY26Δ*acs*-*yjcH*-*actP*, and the complemented FY26C*acs*-*yjcH*-*actP*), and PBS buffer was used as the negative control. As shown in Figure 7E, panels a–d, the peribronchial structure of the lung sections for PBS-inoculated ducks was intact at each time point. The parabronchial lumens and pulmonary alveolus were open and aerated, and almost none inflammatory cells infiltrated around the lumen of PBS-inoculated tissue sections. In contrast to the lungs from the uninfected ducks, we observed the severe pathological changes in the lungs from the FY26-infected ducks (Figure 7E, panels e–h). In the lungs of FY26-challenged duckling (24 hpi), the interval between the pulmonary alveolus was significantly widened, and a large number of lymphocytes infiltrated in the interval. The other lesions contained the exudation in the bronchioles, alveolar congestion, and capillary interstitial congestion in the pulmonary interstitium (Figure 7E, panel f). With the persistence of APEC infection in duck lungs, the highly inflamed and congested areas were observed in the infected lungs at 36 hpi or 48 hpi, accompanied with obvious consolidation of lung tissues (Figure 7E, panels g–h). Deletion of *acs*-*yjcH*-*actP* genes decreased the lesions progress relative to that of FY26-infected ducks (Figure 7E). The relatively light pathological changes and less severe inflammation were present in the lungs from FY26Δ*acs* and FY26Δ*acs*-*yjcH*-*actP* infected ducks (Figure 7E). Infection with the complemented FY26C*acs*-*yjcH*-*actP* caused severe inflammation and pathological changes of the lung at 24 hpi, similar to that caused by wild-type FY26.

These results illustrated that the acetate assimilation system encoded by *acs*-*yjcH*-*actP* operon not only acted as an APEC intracellular survival factor but also a virulence factor during avian infection route. The acetate assimilation system might facilitate APEC to escape from phagocytes clearance of respiratory system and promote its to enter the bloodstream to cause sepsis and multisystemic infection.

## Discussion

APEC/ExPEC holds extraintestinal infection-specific virulence properties, including many known adhesins, invasins, serum resistance factors, iron acquisition systems, typical transcriptional regulators, and etc., which are clearly distinguished from virulence features of IPEC [22, 25, 26]. When APEC/ExPEC infect specific extraintestinal niches, it must evade or escape host immune defense. During ExPEC colonizing human urinary tract, avian respiratory system, and central nervous system, macrophages play critical roles in host defenses to suppress ExPEC infection [4, 52]. However, macrophages fail to clear out highly virulent ExPEC, especially K1 *E. coli*, and ExPEC can spread in the bloodstream to cause septicemia and fatal multisystemic infection [13, 41]. Nowadays, several authors have proposed that APEC/ExPEC is a facultative intracellular pathogens, and more and more evidence reveal that intracellular persistence and replication within macrophages is essential for APEC extraintestinal dissemination [13, 30, 40, 41, 53]. It is well-known that intracellular bacterial life cycle mainly consists of three stages: endocytosis and invasion; persistence and proliferation in the cytosol or PCVs of infected host cells; evasion and recolonization of new host cells. APEC requires intracellular survival factors to maintain the intracellular life cycle during its infection in macrophages. For example, K1 capsule is an important survival factor to promote APEC inhibiting the immune response modulation and resistance to phagolysosomes killing [13, 54]. Until now, there is still no systematic interpretation of APEC intracellular survival/proliferation mechanism, and intracellular survival factors that were especially involved in pathometabolism need to be further revealed.

In this study, we identified that the *acs*-*yjcH*-*actP* operon encoding acetate assimilation system in typical APEC/ExPEC dominant serotypes (O1:K1, O2:K1, and O18:K1) strains presented the host-induced transcription during its infection in macrophages (HD11 and RAW264.7). The phenomenon indicated that this acetate assimilation was activated during APEC infection in macrophages and presented the dominant state relative to acetate dissimilation. More importantly, acetate can be used as the sole carbon source of *E. coli* and other microbiota [17–20]. It seemed that is an inseparable coordination between APEC intracellular survival and expression of acetate assimilation system. The roles of this acetate assimilation system in APEC replication within macrophages were further determined. Recent evidences reveal that the intracellular pathogen can exploit its metabolic pathways to uptake host nutrient sources, aiming to enhance the active survival and proliferation in host intracellular compartments [4, 6, 7, 55]. Lactate dehydrogenases (LDHs) is an important survival factor of *Neisseria gonorrhoeae* to uptake host-derived lactate, which is the critical carbon source for survival/replication of *N. gonorrhoeae* in phagocytic or epithelial cells [56]. *Shigella* can reprogram the central metabolism of infected host cells to produce redundant pyruvate, which acts as a favorable energy source to maintain *Shigella* intracellular growth [2]. The metabolic requirements of APEC proliferation in biofilm-like intracellular bacterial communities (IBCs) during its urinary tract infection, and galactoside metabolism promote UPEC colonization in bladder epithelial cells [5]. Our result showed that acetate assimilation system encoded by *acs*-*yjcH*-*actP* operon acted as a novel intracellular survival factor to promote APEC replication within macrophages. Our research might be the first to unravel the metabolic crosstalk of APEC intracellular survival or replication within macrophages, and acetate metabolic requirement might be an important strategy of APEC/ExPEC pathometabolism. However, the host-induced expression of acetate assimilation system didn’t happen when APEC invasion in non-phagocytic cells, and the *ackA*-*pta* operon encoding the acetate dissimilation system exhibited a low level of transcription during APEC infection. We also perform the in vitro adhesion and invasion assays by non-phagocytic (DF-1) cells, even hemagglutination (HA) assays. But this acetate assimilation system didn’t take roles in APEC internalization (data not shown).

In higher eukaryotes, acetate plays a critical role in supporting energy homeostasis. Exogenous acetate produced by the intestinal commensal microbiota can be assimilated and used by various host metabolic pathways. Besides being a fuel for central metabolism, the emerging studies indicate that acetate might act as a metabolic signal in regulating host gene transcription [17, 49]. For example, acetate promotes the expression of genes, involved in fatty acid oxidation, to suppress body fat accumulation [57]. The effect of acetate assimilation system on host cell damage during APEC interaction with macrophages was assessed. Deletion of *acs*-*yjcH*-*actP* operon in APEC significantly impaired its cytotoxic level to macrophages (HD11 and RAW264.7). Our study showed that acetate assimilation system promoted APEC to damage macrophages, when bacteria simultaneously replicated within macrophages. Recent research indicated that Group A *Streptococcus* (GAS) also simultaneously mediate its cytotoxic damage to human macrophages and cytosolic replication within macrophages, aiming to enhance GAS cell-to-cell spread [58]. The acetate assimilation system might be not only involved in APEC proliferation within macrophages but also promote be APEC evasion/reinfection of new macrophages.

Many pieces of evidences show that acetate produced by intestinal commensal microbiota has beneficial roles in host central energy metabolism and is closely correlated with multiple host physiological features. The acetate is the main component of intestinal short-chain fatty acids (SCFAs), which are the fermentation byproducts produced by the intestinal microbiota. The butyrate and propionate are the other major components of intestinal SCFAs, and three major SCFAs present about 60:25:15 of the molar ratio in the intestinal environment. The accumulating insights of intestinal SCFAs implicate that these byproducts play critical roles in the modulation of host energy metabolism, blood pressure, and inflammatory response, which is beneficial to host energy homeostasis and immunomodulatory functions [17]. However, for the pathogenic microorganisms, SCFAs can be used as an important induction signal, which modulate strongly the expression of virulence-related factors of intestinal pathogenic *E. coli*, such as Shigatoxigenic *E. coli* (STEC) O157:H7 strains. The SCFAs homeostasis (i.e., the steady concentration of SCFAs released by intestinal probiotics) in the intestinal environment might be out of balance by the response activity of pathogenic bacteria to deprive the intestinal microbiota-derived SCFAs [17]. As a typical example, the consumption imbalance of intestinal acetate caused by *Vibrio cholerae* leads to host metabolic disorders and lipid over-accumulation, eventually resulting in host lethality [59]. Liu et al. recently reported that SCFAs can suppress the production of proinflammatory cytokines in lipopolysaccharide (LPS)-stimulated macrophages through inhibiting LPS-induced NF-κB activation [38]. Our qRT-PCR results showed that the transcription of pro-inflammatory cytokines (IL-1β, IL-6, IL-8, IL-12β, and TNF-α) and iNOS were obviously down-regulated in FY26Δ*acs*-*yjcH*-*actP* infected macrophages compared to that in wild-type FY26 infected cells. An important incentive of the successful survival and proliferation during pathogenic bacteria interaction with phagocytosis is the excessive inflammation, accompanied by immune failure and body inflammatory damage [4]. Due to acetate involved in modulation of host inflammatory response [17, 49, 51], our results revealed that the intracellular acetate consumption during facultative intracellular bacteria replication within macrophages might promote immunomodulatory disorders (i.e. imbalance of pro-inflammatory and anti-inflammatory responses), resulting in excessively pro-inflammatory responses of host macrophages. Intracellular acetate, besides serving as an energy source, exploited by APEC/ExPEC during its proliferation in macrophages, as well as a signaling molecule, which was hijacked to avoid or suppress the immune response of macrophages. Whether or how intracellular acetate consumption leads to metabolic disorders of host macrophages will be determined to reveal more intimate host–microbe relationships.

APEC can enter the avian respiratory tract by dissemination of fecal dust, which is the major routine to infect avian extraintestinal niches [44, 52]. When APEC colonizing avian lung tissues, macrophages play critical roles in host defenses to suppress APEC infection [4, 52]. Since the acetate assimilation system encoded by *acs*-*yjcH*-*actP* operon acted as a novel intracellular survival factor to promote APEC replication within macrophages, we further identified its roles in virulence-associated phenotype. Similar to survival in macrophages, the acetate assimilation system was also increasingly expressed during APEC colonization in the lung in vivo for avian colisepticemia models. Deletion of *actP*-*yjcH*-*acs* operon attenuated APEC virulence and colonization capability in avian lungs in vivo, suggesting that loss of this acetate system might impair APEC capability to pass through the immune defense of avian lung tissues. However, deletion of *acs*-*yjcH*-*actP* operon had no effect on the APEC virulence in murine sepsis model and bacteria proliferation in the avian blood in vivo. Our study indicated that the acetate assimilation system acted as a virulence factor and conferred a fitness advantage of APEC early colonization for avian colisepticemia models. Recent reports show that the macrophages of avian lungs fail to clear out highly virulent APEC, which can spread in the bloodstream to cause disease (i.e. avian colibacillosis) [13, 41]. Based on our research, the acetate assimilation system might facilitate APEC escape from phagocytes clearance of respiratory system and promote its entry the bloodstream to cause sepsis and multisystemic infection. Our research implies of the acetate permease (this outer membrane protein encoded by *actP* gene) as a subunit vaccine candidate.

## Additional files

**Additional file 1.** **Bacterial strains and plasmids used in this study.**

**Additional file 2.** **Oligonucleotide sequences used as PCR primers.**

## Abbreviations

ExPEC: extra-intestinal pathogenic *Escherichia coli*
APEC: avian pathogenic *Escherichia coli*
Acs: acetyl-CoA synthetase
LDH: lactate dehydrogenase
NO: nitric oxide
iNOS: nitric oxide synthase
LB: Luria–Bertani
SCFAs: short-chain fatty acids
MOI: multiplicity of infection
