# Supplementary material for: Acetate metabolic requirement of avian pathogenic Escherichia coli promotes its intracellular proliferation within macrophage
Source: Vet Res. 2019 May 2;50:31. doi: 10.1186/s13567-019-0650-2 (PMC6498577; doi:10.1186/s13567-019-0650-2)
Supplement: Supplementary file 1 — Additional file 1. Bacterial strains and plasmids used in this study. [file 13567_2019_650_MOESM1_ESM.docx]

Additional file 1: **Bacterial strains and plasmids used in this study.**

| **Bacterial strains and plasmids** | **Genotype or relevant characteristics** |
| --- | --- |
| **Bacterial strains** |  |
| *E. coli* DH5α | Plasmid propagation strain |
| FY26 | O2:K1; ST 95; phylogroup B2 |
| FY26Δ*acs-yjcH-actP* | *acs-yjcH-actP* operon deletion in FY26 |
| FY26Δ*acs* | *acs* deletion in FY26 |
| FY26Δ*actP* | *actP* deletion in FY26 |
| FY26Δ*satP* | *satP* deletion in FY26 |
| FY26C*acs-yjcH-actP* | FY26Δ*acs-yjcH-actP* with plasmid pSTV28-*acs-yjcH-actP* |
| ***Plasmids*** |  |
| pSTV28 | A medium-copy plasmid |
| pSTV28*-acs-yjcH-actP* | pSTV28 carrying *hlyF* coding region and its putative promoter |
| pKD4 | template for λ-Red Kan^r^ cassette |
| pCP20 | encodes FLP recombinase for removal of resistance cassette |
| pKD46 | λ-Red recombinase expression |
