# Supplementary material for: Acetate metabolic requirement of avian pathogenic Escherichia coli promotes its intracellular proliferation within macrophage
Source: Vet Res. 2019 May 2;50:31. doi: 10.1186/s13567-019-0650-2 (PMC6498577; doi:10.1186/s13567-019-0650-2)
Supplement: Supplementary file 2 — Additional file 2. Oligonucleotide sequences used as PCR primers. [file 13567_2019_650_MOESM2_ESM.docx]

Additional file 2: Oligonucleotide sequences used as PCR primers.

| **Primers** | **Sequence (5'-3')** | |
| --- | --- | --- |
| **General PCR for cloning** |  | |
| pSTV28-*acs-yjcH-actP*-F | CGAGCTCCACGACAGTAACCGCACCTA | |
| pSTV28-*acs-yjcH-actP*-R | CCCAAGCTTGTTCGGTCACTACGCCATTT | |
| pSTV28-*satP* | CGAGCTCAGGTGTGTCGCATTTGCTTTCCA | |
| pSTV28-*satP* | CCCAAGCTTCAAACAGGCGCTGATCCTTAACT | |
| **For Deletion^a^** |  | |
| Del-*acs*-F | TTTAACGCTTATGCCACATATTATTAACATCTTACAAGGAGAACAAAAGCgtgtaggctggagctgcttc | |
| Del-*acs*-R | GTCTTATCAGGCCTACAAACCGTTACCGACTCGCATCCGGCAATTGTGGGcatatgaatatcctccttag | |
| Del-*actP*-F | GGCGAATTCGACCGTCTTAATAACGAAGTCCTGCATGAGGTACAAGCATCgtgtaggctggagctgcttc | |
| Del-*actP*-R | ATGATGTTCTGAAGAAATTCCCCGATCAAGCGACCGGGGAAGGGGAGAGAcatatgaatatcctccttag | |
| Del-*satP*-F | ATTCAGGGAAATTATTTCACCATTCATTCGATGATGATTTTTGAGGAATTgtgtaggctggagctgcttc | |
| Del-*satP*-R | AGCGCACGTTGTTAACAATCTTATCCCTTCGAGCGGGGGGAGCTTAACAAcatatgaatatcctccttag | |
| Del-*acs-yjcH-actP*-F | TTTAACGCTTATGCCACATATTATTAACATCTTACAAGGAGAACAAAAGCgtgtaggctggagctgcttc | |
| Del-*acs-yjcH-actP*-R | ATGATGTTCTGAAGAAATTCCCCGATCAAGCGACCGGGGAAGGGGAGAGAcatatgaatatcctccttag | |
| **For RT-PCR** |  | |
| *acs*-qPCR-F | CGCCTACGTCACGCTTAAT () | |
| *acs*-qPCR-R | AGCGGGCCAATCTCTTTAC | |
| *yjcH*-qPCR-F | GCCACCATCCTGTCGATTATTA | |
| *yjcH*-qPCR-R | CGCTGGTGTTCGGATTCA | |
| *actP*-qPCR-F | TTCTACGCCACCGGATTTATG | |
| *actP*-qPCR-R | CCCGCCGCGTCTTTATATT | |
| *ackA*-qPCR-F | ACTGCCGCTATGTTGAAGAC | |
| *ackA*-qPCR-R | CAGTGTAGGCACCGATGTATTT | |
| *pta*-qPCR-F | CTACCACGCTAACACCAAAGA | |
| *pta*-qPCR-R | AGTTCAGAGACTGGGCAAAC | |
| *satP*-qPCR-F | TTCTGCTGATGCCGAAACT | |
| *satP*-qPCR-R | GCGTGCCGAAGAACATAAAC | |
| *dnaE*-qPCR-F | ATGTCGGAGGCGTAAGGCT | |
| *dnaE* -qPCR-R | TCCAGGGCGTCAGTAAACAA | |
|  | Sequence of primers | Gene accession number |
| *β-actin*-qPCR-F | GAGAAATTGTGCGTGACATCA | NM_205518.1 |
| *β-actin*-qPCR-R | CCTGAACCTCTCATTGCCA |  |
| *IL-1β*-qPCR-F | ATGACCAAACTGCTGCGGAG | AJ245728 |
| *IL-1β*-qPCR-R | GTCGCTGTCAGCAAAGTCCC |  |
| *IL-6*-qPCR-F | CAGGACGAGATGTGCAAGAAG | AJ309540 |
| *IL-6*-qPCR-R | CCCTCACGGTCTTCTCCATA |  |
| *iNOS*-qPCR-F | GCATTCTTATTGGCCCAGGA | U46504 |
| *iNOS*-qPCR-R | CATAGAGACGCTGCTGCCAG |  |
| *IL-8*-qPCR-F | TTGGAAGCCACTTCAGTCAGAC | NM_205498.1 |
| *IL-8*-qPCR-R | GGAGCAGGAGGAATTACCAGTT |  |
| *IL-12α*-qPCR-F | CAACAGTCAGAGCAACACAATAA | AY262751 |
| *IL-12α*-qPCR-R | CTGCAGGCATTTGCTCATATC |  |
| *IL-12β*-qPCR-F | CTGTGGCTCGCACTGATAAA | AJ564201 |
| *IL-12β*-qPCR-R | CAATGACCTCCAGGAACATCTC |  |
| *TNF-α*-qPCR-F | GACAGCCTATGCCAACAAGTA | AY765397.1 |
| *TNF-α*-qPCR-R | TTACAGGAAGGGCAACTCATC |  |
| *IL-4*-qPCR-F | GAGGTTTCCTGCGTCAAGAT | AJ621735 |
| *IL-4*-qPCR-R | GCTGGCTCTCCCAAACAA |  |
| *IL-10*-qPCR-F | AGCTGAGGGTGAAGTTTGAG | AJ621614 |
| *IL-10*-qPCR-R | AACTCATCCAGCAGTTCAGAG |  |
| *IL-13*-qPCR-F | CCGACTGCAAGAAGGACTAC | AJ621735 |
| *IL-13*-qPCR-R | CAAGAAGTTCCGCAGGTAGAT |  |

- Underlined are restriction cutting sites;
- Capital letters represent homologous fragments of the deleted genes.
